# Identification and Analysis of Phenolic Compounds in *Vaccinium uliginosum* L. and Its Lipid-Lowering Activity In Vitro

**DOI:** 10.3390/foods13213438

**Published:** 2024-10-28

**Authors:** Ying Zhang, Wenjiang Dong, Manjun Zhao, Jiyue Zhang, Li Li, Yan Ma, Xianjun Meng, Yuehua Wang

**Affiliations:** 1 College of Food Science, Shenyang Agricultural University, Dongling Road 120, Shenhe District, Shenyang 110866, China; 2 Spice and Beverage Research Institute, Chinese Academy of Tropical Agricultural Sciences, National Center of Important Tropical Crops Engineering and Technology Research, Wanning 571533, China; 3 Center of Experiment Teaching, Shenyang Normal University, Shenyang 110034, China

**Keywords:** *Vaccinium uliginosum* L., polyphenol, anthocyanins, lipid lowering, in vitro

## Abstract

*Vaccinium uliginosum* L. (VU), rich in polyphenols, is an important wild berry resource primarily distributed in extremely cold regions. However, the detailed composition of *Vaccinium uliginosum* L. polyphenols (VUPs) has not been reported, which limits the development and utilization of VU. In this study, VU-free polyphenols (VUFPs) and VU-bound polyphenols (VUBPs) were, respectively, extracted using an ultrasonic, complex enzyme and alkali extraction method; the compositions were identified using ultra-performance liquid chromatography–electrospray ionization mass spectrometry, and lipid-lowering activity in vitro was evaluated. The results showed that 885 polyphenols and 47 anthocyanins were detected in the VUFPs and VUBPs, and 30 anthocyanin monomers were firstly detected in VU. Compared with the model group, the accumulation of lipid droplets and the total cholesterol and triglyceride contents in the high-concentration VUP group reduced by 36.95%, 65.82%, and 62.43%, respectively, and liver damage was also alleviated. It was also found that VUP can regulate the level of *Asialoglycoprotein receptor* 1, a new target for lipid lowering. In summary, this study provides a detailed report on VUP for the first time, confirming that VUP has lipid-lowering potential in vitro. These findings suggest new strategies and theoretical support for the development and utilization of VU, especially in the field of functional foods.

## 1. Introduction

Hyperlipidemia is a common metabolic disease that typically refers to elevated levels of lipids in the bloodstream, characterized by higher-than-normal levels of total cholesterol (TC) and triglyceride (TG). Hyperlipidemia is closely linked to metabolic diseases like insulin resistance, diabetes, and fatty liver [1,2]. This also easily causes atherosclerosis, coronary heart disease, and hypertension [3,4]. Currently, statins are the most commonly used lipid-lowering drugs [5]. However, lipid-lowering drugs may cause abnormalities, such as abnormal liver function and increased aspartate aminotransferase (AST) and alanine aminotransferase (ALT) levels [6]. Given the adverse effects associated with synthetic lipid-lowering medications, there is a critical unmet need for identifying natural alternatives with potent efficacy and minimal side effects. Other researchers have recently focused on the effects of natural bioactive compounds, such as polyphenols and flavonoids, on hyperlipidemia and related diseases [7,8,9].

Polyphenols, plant-derived compounds, are known for their health-promoting effects, including antioxidant, anti-inflammatory, vision-protective, and constipation-relieving properties [10,11,12,13]. Anthocyanin is a water-soluble pigment that contributes to the bright colors of plant organs. In its basic structure, anthocyanidin is linked to a sugar molecule (such as glucoside, arabinoside, or xyloside) through a glycosidic bond [14]. There are several common anthocyanidins: Cyanidin, Delphinidin, Malvidin, Pelargonidin, Peonidin, and Petunidin. Of the six most common forms, Malvidin is the most stable, and Delphinidin is the least stable [15]. Many studies have found that the content of blueberry polyphenols is very high, especially in wild blueberry [16,17].

*Vaccinium uliginosum* L. (VU) is a shrub plant belonging to the genus *Vaccinium* of the family Ericaceae [18]. Its fruits are small, blue-purple berries, which are wild blueberries. VU is indigenous to the high altitudes of Asia, North America, and Europe [19]. In China, the distribution of VU is predominantly concentrated in the Xing’an region and in the Changbai Mountains [20]. As early as 1996, researchers compared the morphology and biostatistics of VU in Spain, France, and parts of Eastern Europe [21]. Like cultivated blueberries, VU is a rich source of dietary bioactive compounds, including vitamin C, flavonoids, and phenolic compounds [22,23,24].

Despite the recognized health benefits of VU, the comprehensive analysis of its polyphenolic composition and specific lipid-lowering mechanisms has not been conducted. This gap in knowledge hinders the potential therapeutic application and broader utilization of VU. A new lipid-lowering target, ASGR1, has been found [25], but whether polyphenols can be associated with it has not been studied.

The purpose of this study is to explore the functional components of VU, identify previously undetected anthocyanin monomers, and investigate the lipid-lowering activity of VU polyphenols (VUPs) in vitro. The results address the current gap in knowledge and potentially unlock new applications for VU in functional foods.

## 2. Materials and Methods

### 2.1. Chemicals

Anhydrous ethanol and related reagents were purchased from Tianjin Zhongtian Chemical Co., Ltd. (Tianjin, China). Gallic acid, Folin phenol reagent, AB-8 macroporous resin, and cellulase were purchased from Shanghai Macklin Biochemical Technology Co., Ltd. (Shanghai, China). Oil red O, hematoxylin, and penicillin–streptomycin–gentamicin mixed solution (100 × three antibodies) were purchased from Solarbio Technology Co., Ltd. (Beijing, China). Bovine serum albumin (BSA), magnetic tape terminal (MTT), and Pancreatic Enzymes were purchased from Genview (America). Fetal bovine serum (FBS) was purchased from Zhejiang Tianhang Biotechnology Co., Ltd. (Huzhou, China). Dulbecco’s Modified Eagle Medium (DMEM) was purchased from Gibco (Suzhou, China). The HepG2 cell line was provided by College of Food Science, Shenyang Agricultural University.

### 2.2. Plant Materials

VU was purchased from Yichun City, Heilongjiang Province, in August 2023 and preserved at −20 °C.

### 2.3. Extraction and Purification of VUPs

#### 2.3.1. Extraction of VU-Free Polyphenols (VUFPs)

An ultrasonic compound enzyme method optimized in the laboratory was used to extract the VUFPs. The procedure was as follows: We weighed 50 g of fruit, used a juicer to crush it, added a 5-times-higher volume of 65% ethanol aqueous solution, and then added 0.2% composite enzyme (pectinase–cellulase = 1:1). The mixture was subjected to enzymatic hydrolysis at 50 °C for 2 h, and then an ultrasound at 40 °C with 300 W for 50 min. The solution was centrifuged at 10,000 rpm for 5 min (5804R, Eppendorf, Hamburg, Germany), and the supernatant was taken and concentrated.

#### 2.3.2. Extraction of VU-Bound Polyphenols (VUBPs)

The VUBPs were extracted by alkali extraction based on the previous experiments. The centrifuged pomace from Section 2.3.1 was added to 3 mol/L NaOH solution at a ratio of 1:5, shaken at 37 °C for 4 h, and then sonicated at 40 °C with 300 W for 50 min. The pH was adjusted to 7.0, and the mixture was centrifuged at 10,000 rpm for 5 min to obtain the supernatant. The supernatant was extracted with ethyl acetate, and the operation was repeated three times, and then rotary-evaporated to obtain the test solution of VUBPs.

AB-8 resin was used to purify the VUFPs and VUBPs [26]. The samples underwent freeze-drying and were subsequently preserved in a −20 °C, light-shielded setting.

### 2.4. Folin Phenol Method to Determine Total Phenol Content (TPC)

The Folin phenol method was used to determine the TPC, and the reference method was slightly modified [27]. We took 1 mL sample, added 1 mL of Folin phenol reagent, and let it stand for 5 min. Then, we added 5 mL of 7.5% sodium carbonate aqueous solution, shook it well, and let it stand for 2 h to react fully. Absorbance was measured using a ultraviolet-visible spectrophotometer (Evolution201, Thermo Fisher Scientific, Waltham, MA, USA) at a detection wavelength of 760 nm.

### 2.5. The pH Differential Method to Determine Total Anthocyanin Content (TAC)

The pH differential method was used to determine the TAC [28]. Two 1 mL samples were added to the test tubes, and buffer solutions with pH = 1.0 and pH = 4.5 were added, respectively.

After the samples were placed in the dark for 1 h, absorbance was measured at 520 nm and 700 nm.

$$TAC=\frac{\left[\left(A520-A700\right)pH=1.0-\left(A520-A700\right)pH=4.5\right]\times MW\times DF\times 100}{\varepsilon \times L\times m}$$

The results are expressed as mg/100 mL, where A, absorbance value; MW, the relative molecular weight of Cyanidin-3-*O*-glucoside (C3G) (449); DF, dilution factor; ε, the molar extinction coefficient of C3G (26,900); L, the length of the optical path (1 cm); and m, sample mass.

### 2.6. Determination of Antioxidant Activity

We referred to the following method to determine the scavenging rate of 2,2-diphenyl-1-picrylhydrazyl (DPPH), but we modified it [29]. A total of 100 μL of 0.2 mM DPPH ethanol solution was added to each well in a 96-well plate. Subsequently, 100 μL of VUFPs and VUBPs (0.2 mg/mL) was added to the respective samples to be tested. The samples were kept in the dark for 30 min. The absorbance value was measured at 517 nm, and the DPPH scavenging rate was calculated.

$$DPPH\;scavenging\;rate\left(\%\right)=\left(1-\frac{S-S_{0}}{C-C_{0}}\right)\times 100\%$$

where S, sample + DPPH; S_0_, sample + absolute ethanol; C, absolute ethanol + DPPH; and C_0_, absolute ethanol.

The ferric reducing antioxidant power (FRAP) was assessed utilizing a kit (S0116, Beyotime, Shanghai, China).

### 2.7. Identification of Polyphenols

The analysis of the samples was conducted utilizing an ultra-performance liquid chromatography–electrospray ionization mass spectrometry (UPLC-ESI-MS/MS) system (UPLC, ExionLC™ AD (AB SCIEX, Singapore), https://sciex.com.cn/, accessed on 28 February 2024; MS/MS: Applied Biosystems 6500 Triple Quadrupole (AB SCIEX, Singapore), https://sciex.com.cn/, accessed on 28 February 2024) [30,31].

The UPLC conditions included an Agilent SB-C18 column (1.8 µm, 2.1 mm × 100 mm), with mobile phase A being pure water with 0.1% formic acid and mobile phase B being acetonitrile with 0.1% formic acid. The samples were eluted using gradient elution, with a flow velocity of 0.35 mL/min, the column oven set to 40 °C, and an injection volume of 2 μL. The effluent was connected to an ESI triple quadrupole–linear ion trap (QTRAP) MS.

The ESI source operated at 550 °C, and the ion spray voltage (IS) was adjusted to 5500 V (positive mode) and −4500 V (negative mode). The gas flow was set at 50 psi (GSI), 60 psi (GSII), or 25 psi (CUR), and the collision-induced dissociation (CID) parameter was configured at a high level. Multiple reaction monitoring (MRM) mode was employed for triple quadrupole (QQQ) scans utilizing nitrogen as the collision gas at a medium flow rate. Further optimization was conducted by refining the declustering potential (DP) and collision energy (CE) settings.

### 2.8. Qualitative and Quantitative Analyses of Monomeric Anthocyanins

The sample extract analysis system is the same as that in Section 2.7.

The UPLC conditions included utilized a Waters ACQUITY BEH C18 column (1.7 µm, 2.1 mm × 100 mm) paired with a solvent system comprising water containing 0.1% formic acid and methanol also fortified with 0.1% formic acid. After gradient elution, it stood for 2 min; the flow rate, temperature, and injection volume were the same as those in Section 2.7.

The ESI temperature was maintained at 550 °C, with a mass spectrometry voltage of 5500 V in positive ion mode, and the CUR gas flow was set to 35 psi. Using the Q-Trap 6500+ system, each ion pair was scanned and detected with optimized DP and CE settings.

### 2.9. Cell Culture and Toxicity Test

HepG2 cells were cultured in DMEM (10% FBS, 1% three antibodies) at 37 °C and 5% CO_2_.

The cytotoxicity of the VUPs was determined using an MTT assay. HepG2 cells were seeded in 96-well plates at a density of 7 × 10^4^ cells and cultured for 24 h. Concentrations of 0, 1, 1.5, 2, 2.5, and 3 mg/mL of VUPs were added. After being cultured for 24 h, 20 μL MTT reagent (5 mg/mL) was added and cultured for 4 h. Subsequent to discarding the supernatant, 150 μL of dimethyl sulfoxide solution was introduced. After 10 min, the absorbance value was measured at 570 nm, with 0 mg/mL as the control, followed by the calculation of cell viability.

### 2.10. Establishment and Administration of High-Fat Cell Model

HepG2 cells were cultured for 24 h. Oleic acid (OA)–BSA solution (0.5 mmol/L OA mixed with 10% BSA) was used to induce lipid accumulation in the cells (as the OA group), while the control group was treated with 10% BSA. After 24 h, different concentrations of VUPs were added to the treatment group and incubated for 24 h.

### 2.11. Oil Red O Staining and OD Value Determination

Oil red O staining was performed in reference to this modified method [32]. The supernatant was discarded, and residue was washed away with PBS. The cells were fixed with 4% formaldehyde fixative (1 mL) for 30 min. After discarding the fixative, cells were stained with 1 mL of oil red O solution for 10 min. Oil red O dye solution was sucked out and washed with 60% isopropanol. The nucleus was re-stained with hematoxylin for 2 min. Distilled water was washed to distinguish between the blue and red parts and observed and photographed under a light microscope (AE2000, MOTIC, Shenzhen, China).

The photographed samples were dissolved in 200 μL isopropanol for 15 min, and absorbance was measured at 510 nm.

### 2.12. Metabolic Index Determination

The levels of TC, TG, aspartate aminotransferase (AST), and alanine aminotransferase (ALT) in the cells were measured using kits purchased from Nanjing JianCheng Bioengineering Institute (Nanjing, China) following the product manual. The kits used were a TC kit (A111-1-1), a TG kit (A110-1-1), an AST kit (C010-2-1), and an ALT kit (C009-2-1).

### 2.13. Enzyme-Linked Immunosorbent Assay (ELISA)

*Asialoglycoprotein receptor* 1 (ASGR1) was examined using the ASGR1 Kit (YJ340325, Shanghai Enzyme-linked Biotechnology Co., Ltd., Shanghai, China) following the product manual.

### 2.14. Statistical Analysis

In this study, all the data were analyzed using GraphPad Prism 8.0 (GraphPad Software Inc., San Diego, CA, USA). The results are expressed as mean ± standard deviation (SD). Comparisons between the groups were analyzed using one-way ANOVA, with *p* < 0.05 considered a statistically significant difference.

## 3. Results

### 3.1. The TPC and TAC of VUFPs and VUBPs

The TPC and TAC in the VUFPs and VUBPs are shown in Table 1. Table 1 shows that the contents of TPC and TAC in the VUFPs are significantly higher than those in the VUBPs. TAC was not even detected in the VUBPs. It may be that the anthocyanin content in VUBPs is too low and not within the detection range.

### 3.2. Antioxidant Activity of VUFPs and VUBPs

Antioxidant capacity is one of the important indexes of food quality. By measuring the antioxidant capacity, we can screen out the ingredients with higher antioxidant activity. As shown in Table 1, the VUFPs had a DPPH scavenging rate of 87.32 ± 3.21%, whereas the VUBPs had a DPPH scavenging rate of only 10.02 ± 0.5%. The DPPH scavenging rate was significantly higher in the VUFPs than in it was in the VUBPs (*p* < 0.001). Furthermore, the VUFPs exhibited significantly more antioxidant activity than the VUBPs.

The total antioxidant capacity measured according to the FRAP method is shown in Table 1. Specifically, the total antioxidant capacity of the VUFPs was 3313.41 ± 178.26 mmol/mg, while that of the VUBPs was 24.62 ± 1.78 mmol/mg. Based on these data, we can see that the antioxidant capacity of the VUFPs is more than 100 times higher than that of the VUBPs.

In summary, it can be seen that the VUFPs exhibited a stronger antioxidant capacity compared to that of the VUBPs.

### 3.3. Polyphenol Metabolomics

**Figure 1:**
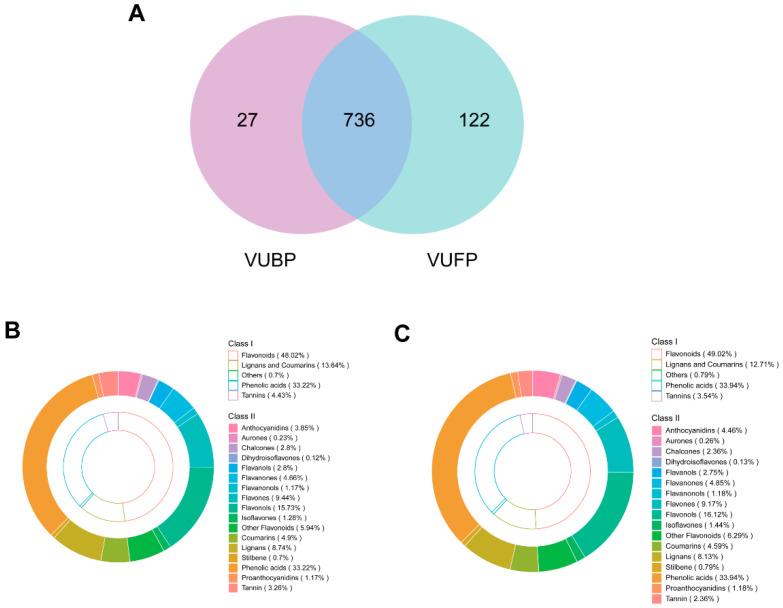
The classification of polyphenol metabolites. (**A**) The number of polyphenol metabolites in the VUFPs and VUBPs; (**B**) VUFP polyphenol metabolites classification; (**C**) VUBP polyphenol metabolites classification.

UPLC-ESI-MS/MS was used to detect the components of the VUFPs and VUBPs. MRM detection in VUFPs and VUBPs multimodal maps is shown in Figure S1. A total of 885 polyphenol metabolites were detected according on the UPLC-ESI-MS/MS detection platform and the self-constructed database (Table S1). Their primary and secondary classifications are indicated in Figure 1. As is obvious from Figure 1, 858 polyphenol metabolites were detected in the VUFPs, and 763 polyphenol metabolites were detected in the VUBPs. In general, polyphenol metabolites can be divided into five categories: phenolic acids, flavonoids, lignans and Coumarins, tannins, and others. From a macro perspective, the compositions of polyphenol content in the VUBP and VUFP groups are similar, but the proportions of the various polyphenol subclasses within these groups differ when classified into primary and secondary categories. According to Class I, the contents of flavonoids (48.02%), others (0.7%), and phenolic acids (33.22%) in the VUFPs are lower than those in the VUBPs. In contrast, the contents of lignans and Coumarins (13.64%) and tannins (4.43%) in the VUFPs are higher than those in the VUBPs. Despite these differences in content, flavonoids and phenolic acids remain the primary phenols in both the polyphenol groups, followed by lignans and Coumarins.

### 3.4. Anthocyanin Monomer Analysis

**Figure 2:**
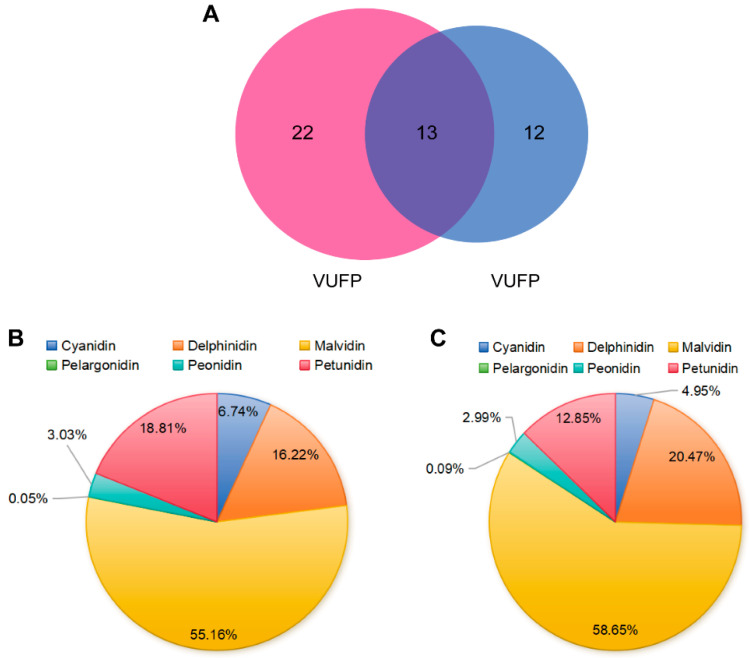
Analysis of anthocyanin monomers in VUFPs and VUBPs. (**A**) Number of anthocyanin monomers in VUFPs and VUBPs; (**B**) VUFP anthocyanin monomer classification; (**C**) VUBP anthocyanin monomer classification.

To further identify the components of the VUPs, UPLC-ESI-MS/MS was used to identify the anthocyanin monomers in the VUFPs and VUBPs. As shown in Figure 2A, 35 anthocyanin monomers were detected in the VUFPs, while 25 anthocyanin monomers were detected in the VUBPs. Among these, 13 anthocyanin monomers were common to both the VUFPs and VUBPs. Additionally, 22 anthocyanin monomers were detected only in the VUFPs, and 12 anthocyanin monomers were detected only in the VUBPs. The specific anthocyanin monomer substances detected are shown in Table S2. From Table S2, it can be seen that the total anthocyanin content detected in the VUFPs was 3837.90 μg/g. Among these, Malvidin had the highest content, accounting for 55.16% of the total anthocyanins detected, followed by Petunidin and Delphinidin, which accounted for 18.81% and 16.22%. The content of Pelargonidin was 1.745 μg/g, accounting for only 0.05% of the total anthocyanins. The total anthocyanin content detected in the VUBPs was extremely low at only 56.33 μg/g. In summary, the content of total anthocyanins in the VUFPs was higher than that in the VUBPs.

### 3.5. Effect of VUPs on the Viability of HepG2 Cells

**Figure 3:**
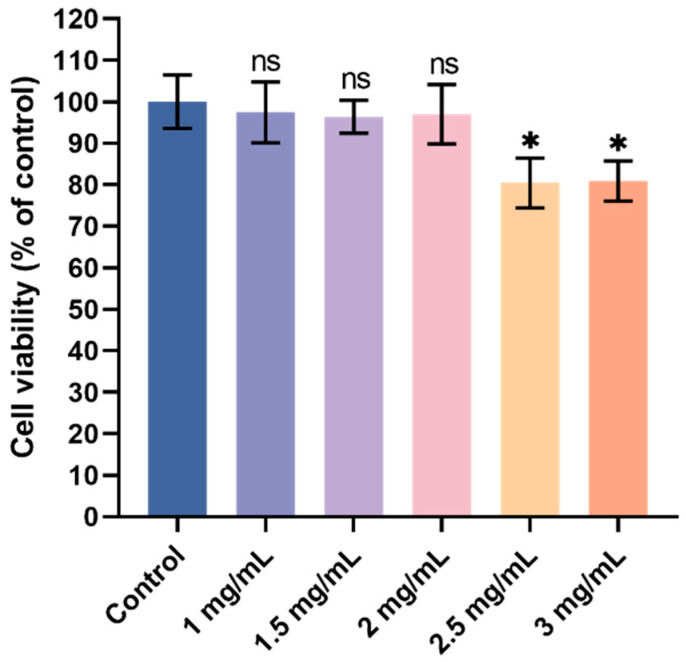
Cell viability (% of control). * *p* < 0.05 compared with control group; ns = not significant.

An MTT assay was used to determine VUPs’ toxic effects on HepG2 cells, and the results are shown in Figure 3. From the cell viability data, 1 mg/mL VUP, 1.5 mg/mL VUP, and 2 mg/mL VUP had no significant difference in the viability of HepG2 cells compared with that of the control group (0 mg/mL VUP). The viability of HepG2 cells (*p* < 0.05) was more significantly reduced by 2.5 mg/mL VUP and 3 mg/mL VUP compared to that of the control. In summary, 1 mg/mL was selected as the low-dose group of VUPs (VUP-L), 1.5 mg/mL as the middle-dose group of VUPs (VUP-M), and 2 mg/mL as the high-dose group of VUPs (VUP-H). These concentrations of VUP had no effect of toxicity on the HepG2 cells.

### 3.6. Effect of VUPs on Lipid Accumulation

**Figure 4:**
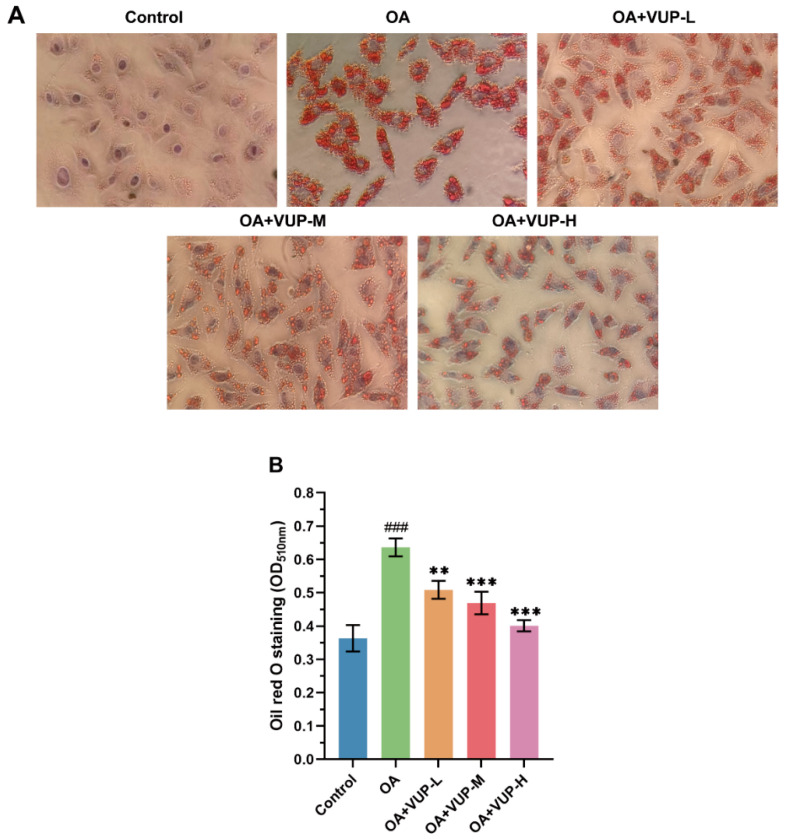
Oil red O staining results. (**A**) Oil red O photography (×400). (**B**) Lipid accumulation at 510 nm. ### *p* < 0.001 compared with control group; ** *p* < 0.01 compared with OA group; *** *p* < 0.001 compared with OA group.

To verify the effect of VUPs on intracellular lipid accumulation, oil red O staining was used to determine intracellular lipid accumulation. From Figure 4A, the control group had no obvious red lipid droplet accumulation. In the OA group, induced by OA, many red lipid droplets were observed to accumulate. Compared with the OA group, the accumulation of red lipid droplets in the treatment group was reduced. The OD value of oil red O staining is shown in Figure 4B; it can also be seen that OA significantly increased the accumulation of lipid droplets in the HepG2 cells (*p* < 0.001). Compared with the OA group, the OA+VUP-L group had reduced lipid accumulation (*p* < 0.01), and both the OA+VUP-M group and the OA+VUP-H group showed extremely significantly reduced considerably lipid accumulation (*p* < 0.001). The results showed that the VUPs could reduce the accumulation of lipid droplets by 20.03%–36.95%.

### 3.7. Effect of VUPs on Lipid Metabolism Indicators

**Figure 5:**
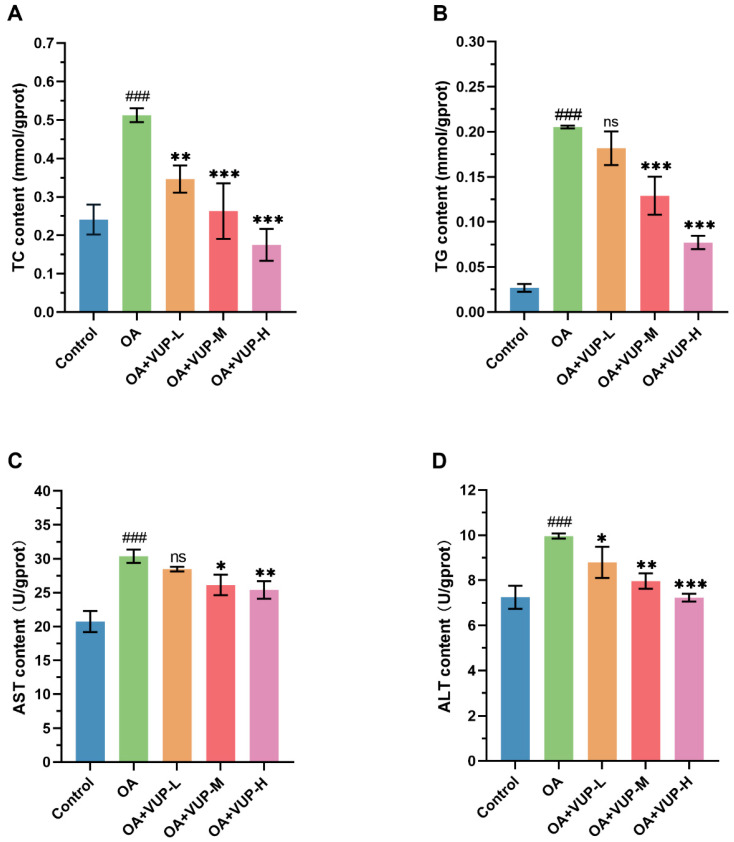
The results of the lipid metabolism and liver injury indicators. (**A**) The change in TC content. (**B**) The change in TG content. (**C**) The change in AST content. (**D**) The change in ALT content. ### *p* < 0.001 compared with the control group; * *p* < 0.05 compared with the OA group; ** *p* < 0.01 compared with the OA group; *** *p* < 0.001 compared with the OA group; ns = not significant.

To explore the effect of VUPs on regulating the lipid levels, we measured the changes in TC and TG contents and the lipid metabolism indicators, and the results are shown in Figure 5A,B. Figure 5A shows the change in TC content. Compared with the control group, the OA group had a significantly increased TC content (*p* < 0.001). Compared with the OA group, the OA+VUP-L group had a significantly reduced TC content (*p* < 0.01), while the OA+VUP-M group and the OA+VUP-H group had significantly reduced TC contents (*p* < 0.001). The change in TG content is shown in Figure 5B, and its trend is similar to that of the TC content. However, unlike the change in TC content, there was no significant difference in TG content between the OA+VUP-L group and the OA group (*p* > 0.05). VUPs can mitigate the increase in TC and TG contents caused by OA (TC and TG were reduced by 32.44–65.82% and 11.45–62.43%). The results showed that the VUPs exhibited lipid-lowering effects.

### 3.8. Effect of VUPs on Liver Function Indicators

The changes in AST and ALT levels were detected to determine the effect of VUPs on liver injury. The effect of the VUPs on the liver function indicators AST and ALT is shown in Figure 5C,D. Compared with the control group, the OA group had significantly increased levels of AST and ALT (*p* < 0.001), indicating increased liver damage. After the VUP treatment, the trend of liver damage caused by OA was improved.

### 3.9. Effect of VUPs on ASGR1 Level

**Figure 6:**
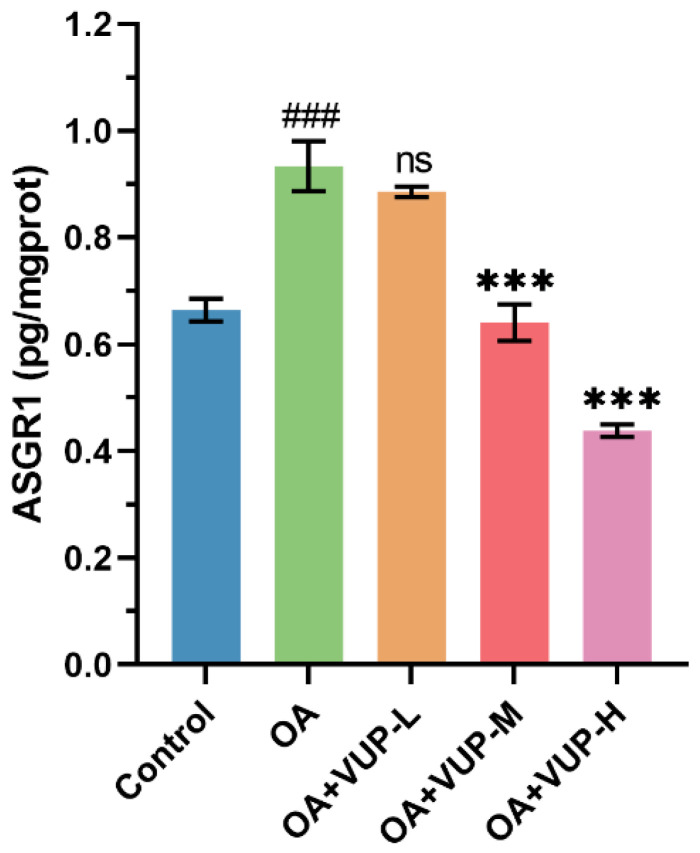
The ASGR1 expression level. ### *p* < 0.001 compared with the control group; *** *p* < 0.001 compared with the OA group; ns = not significant.

To study the regulatory effect of the VUPs on ASGR1, a kit was used to detect the content of ASGR1, and the results are shown in Figure 6. Compared with the control group, the OA group had a significantly increased expression level of ASGR1 in the HepG2 cells (*p* < 0.001). The OA+VUP-L group showed no significant difference in the ASGR1 expression level compared with that of the OA group (*p* > 0.05). However, compared with the OA group, the expression level of ASGR1 was significantly decreased in both the OA+VUP-M group and the OA+VUP-H group (*p* < 0.001). The trend of change in ASGR1 expression level was similar to that of the TC content, as shown in Figure 5A. Notably, the TC content and the ASGR1 expression level in the OA+VUP-H group were lower than those in the control group. The results showed that the VUPs could regulate the level of ASGR1.

## 4. Discussion

The functional composition of VU as a nutrient-rich wild berry has not been thoroughly evaluated. As far as we know, there are no data on the detailed composition of VUPs. The existing studies have reported only the main active components of VU [23,24]. This study aims to fill this knowledge gap by comprehensively extracting VUPs and identifying their detailed components. in addition to the potential for the lipid-lowering activity of VUPs and its possible mechanism.

In this study, a total of 885 polyphenols were detected in VU. To our knowledge, this is the first detailed report identifying the VUFP and VUBP contents. VU is rich in polyphenols. Phenolic acids, flavonoids, lignans, and stilbenes are the most naturally occurring classes of compound [33]. In this study, the polyphenols were divided into five categories. Among them, flavonoids were the most abundant polyphenols. Flavonols are a relatively abundant flavonoid subclass with superior beneficial effects in regulating glycolipid metabolism. Another study found that isorhamnetin, a flavonol, is a relatively stable substance [34], which may be the reason for its high values in this study.

The anthocyanin monomers were further investigated using UPLC-ESI-MS/MS. The results showed that 35 anthocyanin monomers were detected in the VUFPs, and 25 anthocyanin monomers were detected in the VUBPs. Among these anthocyanin monomers, 30 anthocyanin monomers were detected in VU for the first time (as listed in Table S2: numbers 1–4, 6, 9, 11, 13–16, 18, 20, 22, 24–25, 27–28, 30–35, 37, 40, and 42–45). The previous studies have shown that the most abundant anthocyanins detected in VU were Malvidin, followed by Delphinidin, Petunidin, Cyanidin, and Peonidin [22,24,35]. The results of this study were consistent with the previous studies. Malvidin accounts for more than 55% of the total anthocyanins in the VUFPs and the VUBPs. The difference is that Pelargonidin was detected in the VUPs in this study. To our knowledge, this is the first time that Pelargonidin has been detected in VU. The anthocyanin glycosides were mainly glucosides, followed by galactoside, arabinoside, and xyloside [15,36]. In this study, the binding of anthocyanin to sambubioside was detected for the first time in VU, and it only exists in VUFPs. The results of this study suggest that VU may have higher nutritional value, which provides more options and possibilities for the development of VU and healthy products.

VU, rich in polyphenols, has been studied for its functions. Research shows VU’s anthocyanins reduce mouse retinal cell apoptosis and oxidative stress damage [11], inhibit UV-B-induced collagen destruction and inflammation [37], and potentially enhance memory and learning [38]. VUPs exhibit superior functional activities.

To study the lipid-lowering effect of VUPs, this study used HepG2 cells to establish an in vitro high-fat model to verify their lipid-lowering effects. Consistent with the previous studies [29], in the present study, red lipid particles accumulated in the OA-treated HepG2 cells, and the levels of TC and TG were significantly elevated. The results showed that VUPs could reduce lipid accumulation and reduce the contents of TC and TG. Consistent with this study, Zhang et al. found that extracts from Que Zui tea are rich in polyphenols, which can significantly reduce the levels of TC and TG and decrease liver lipid accumulation [39].

An increase in lipid levels can lead to liver injury, fatty liver, and so on [40]. AST and ALT are aminotransferases in the cytoplasm and are primarily involved in the metabolic process of amino acids [41]. AST is mainly found in the liver, muscles, heart, and other tissues, whereas ALT is predominantly present in the liver. Thus, AST and ALT are commonly used to evaluate liver function. When liver cells are damaged, these two enzymes are released into the bloodstream, so an increase in their serum levels may indicate liver damage [42]. In this study, OA caused damage to the HepG2 cells, and we found that the VUPs could reduce the increase in AST and ALT caused by OA. This suggests that VUPs have a protective effect on liver injury. Similarly, Liu et al. found that the polyphenol content of blueberry extract was 52.7%, which had a preventive effect on liver injury [43], and Huang et al. found that tea residue dietary fiber combined with polyphenols can improve liver injury [44].

ASGR1 is a receptor protein primarily present on the surface of hepatocytes [45]. ASGR1 is mainly used to bind and internalize galactose-rich glycoproteins, and it helps to maintain the body’s glycoprotein balance, and also participates in the body’s clearance of exogenous substances. In 2016, a large-scale human genetic study linked ASGR1 to lipid lowering [46], and the results suggest that ASGR1 gene mutations that exhibit a loss of function are linked to lower cholesterol levels and CVD risk. In 2022, a study found that inhibiting the expression of hepatocyte membrane protein ASGR1 can inhibit adipogenesis and promote cholesterol efflux into bile, further excreting the body through feces, thereby reducing the blood and liver lipid levels [25]. Other studies have shown that ASGR1 is associated with lipid lowering [47]. Whether polyphenols can regulate the lipid metabolism through the ASGR1 pathway has not been studied. This study found that VUPs can reduce the increase in ASGR1 levels. Based on the results, we speculate that VUPs achieve their lipid-lowering effect probably by regulating the ASGR1 pathway.

## 5. Conclusions

This study investigated the composition of polyphenols derived from VU and its lipid-lowering effect in vitro. The VUFPs and VUBPs were obtained using different extraction methods. A total of 885 polyphenols and 47 anthocyanins were found, with 30 anthocyanin monomers detected in VU for the first time. It has been shown that VUFPs have a higher content of active substances and exhibit a stronger antioxidant capacity than that of VUBPs. The cell experiments showed that VUPs could effectively reduce the accumulation of cell lipid droplets, reduce the levels of TC and TG, alleviate liver injury, and regulate the expression of lipid-lowering-related protein ASGR1. This study revealed the lipid-lowering potential of VUPs, which may contribute to VU’s development in functional foods and provide a possible new raw material for lipid-lowering drugs.

The detailed compositions and lipid-lowering activity of VUPs were reported in the present study; however, how the identified polyphenols might contribute to the observed lipid-lowering effects is still unclear. According to the above results and discussions, we speculated that VUPs achieve a lipid-lowering effect probably by regulating the ASGR1 pathway, which is worthy of further investigation.

## Figures and Tables

**Table 1:** The TPC, TAC, and antioxidant activity of the VUFPs and VUBPs.

	TPC (mg/100 g FW)	TAC (mg/100 mL FW)	DPPH (%)	FRAP (mmol/mg)
VUFPs	519.95 ± 4.56	68.77 ± 0.96	87.32 ± 3.21	3313.41 ± 178.26
VUBPs	207.37 ± 10.91 ***	-	10.02 ± 0.5 ***	24.62 ± 1.78 ***

Note: “-” means not detected; *** *p* < 0.001 compared with the VUFPs. The abbreviations in this table are as follows: TPC, total phenol content; TAC, total anthocyanin content; DPPH, 2,2-diphenyl-1-picrylhydrazyl; FRAP, ferric reducing antioxidant power; VUFP, *Vaccinium uliginosum* L.-free polyphenol; VUBP, *Vaccinium uliginosum* L.-bound polyphenol.

## Data Availability

The original contributions presented in this study are included in the article/Supplementary Material; further inquiries should be directed to the corresponding author.

## Supplementary Materials

The following supporting information can be downloaded at: https://www.mdpi.com/article/10.3390/foods13213438/s1, Figure S1. MRM detection of multimodal maps; Table S1. VUFPs and VUBPs contents; Table S2. Anthocyanin monomeric substances in VUFPs and VUBPs.
