# Supplementary material for: Identification and Analysis of Phenolic Compounds in Vaccinium uliginosum L. and Its Lipid-Lowering Activity In Vitro
Source: Foods. 2024 Oct 28;13(21):3438. doi: 10.3390/foods13213438 (PMC11545093; doi:10.3390/foods13213438)
Supplement: Supplementary file 1 [file foods-13-03438-s001.zip › Supplementary data Figure S1.pdf]

(A)

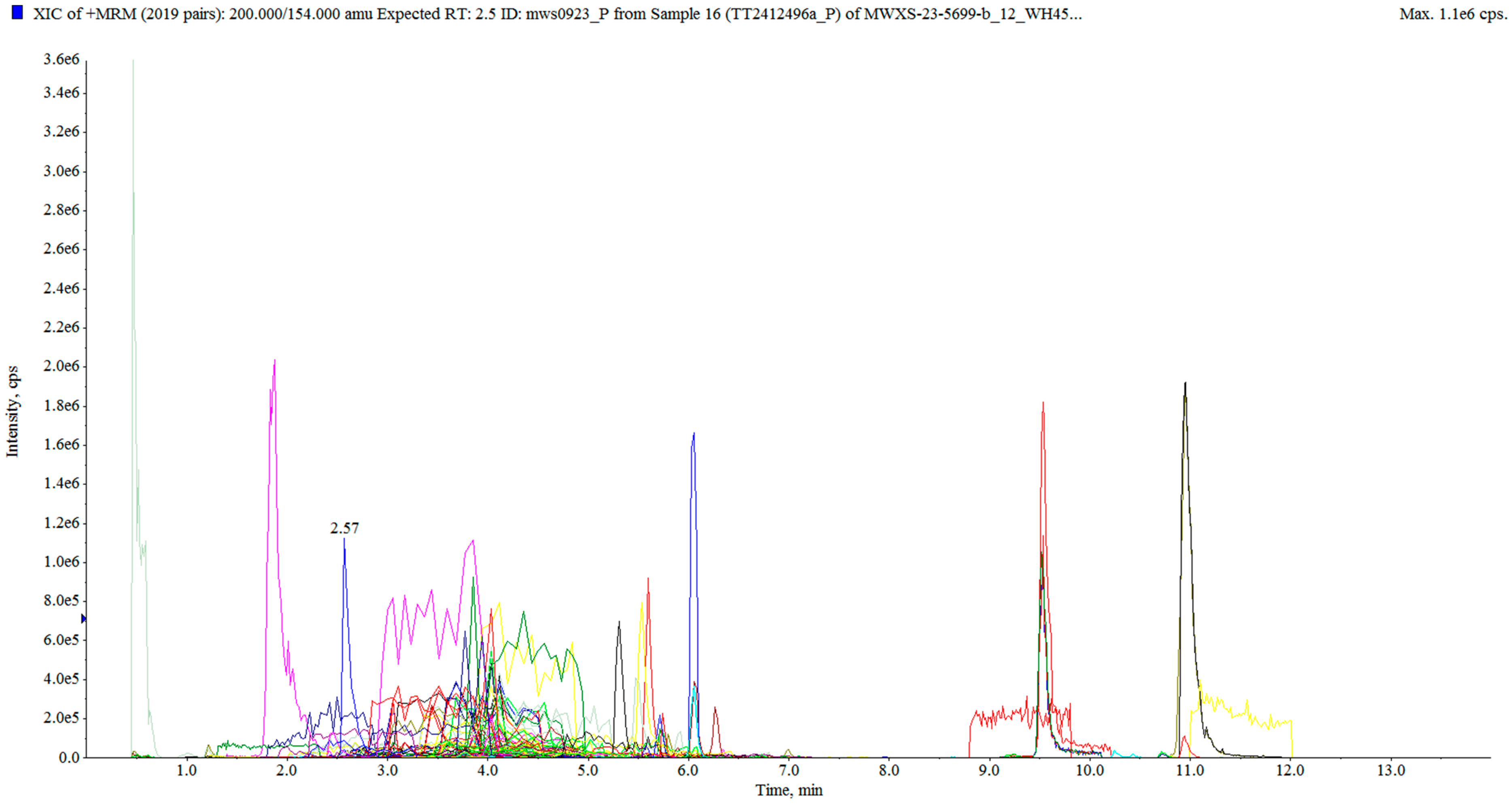
XIC of +MRM (2019 pairs): 200.000/154.000 amu Expected RT: 2.5 ID: mws0923_P from Sample 16 (TT2412496a_P) of MWXS-23-5699-b_12_WH45...
Max. 1.1e6 cps.
Intensity, cps
3.6e6
3.4e6
3.2e6
3.0e6
2.8e6
2.6e6
2.4e6
2.2e6
2.0e6
1.8e6
1.6e6
1.4e6
1.2e6
1.0e6
8.0e5
6.0e5
4.0e5
2.0e5
0.0
2.57
1.0
2.0
3.0
4.0
5.0
6.0
7.0
8.0
9.0
10.0
11.0
12.0
13.0
Time, min

(B)

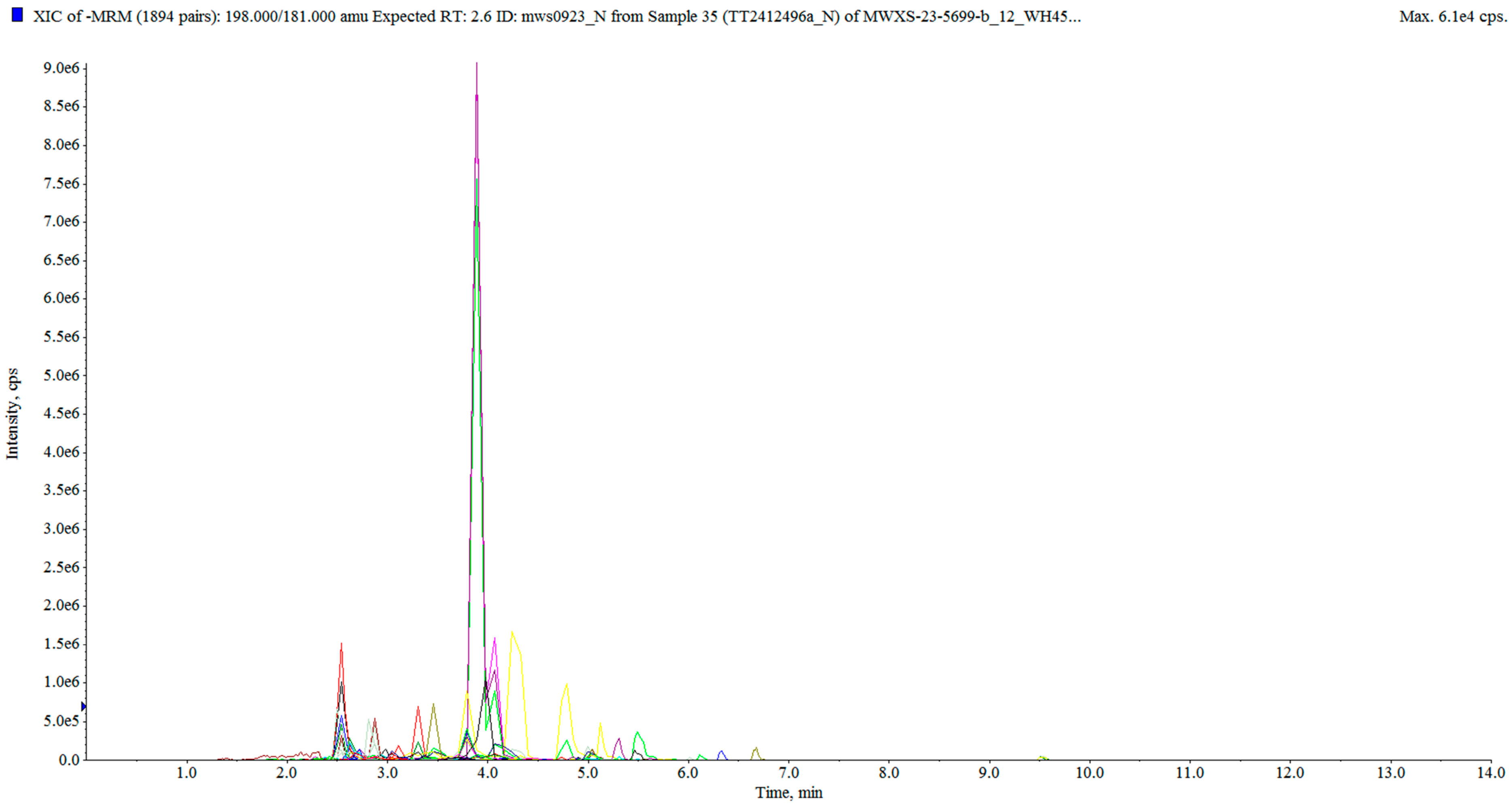
XIC of -MRM (1894 pairs): 198.000/181.000 amu Expected RT: 2.6 ID: mws0923_N from Sample 35 (TT2412496a_N) of MWXS-23-5699-b_12_WH45...
Max. 6.1e4 cps.
Intensity, cps
9.0e6
8.5e6
8.0e6
7.5e6
7.0e6
6.5e6
6.0e6
5.5e6
5.0e6
4.5e6
4.0e6
3.5e6
3.0e6
2.5e6
2.0e6
1.5e6
1.0e6
5.0e5
0.0
1.0
2.0
3.0
4.0
5.0
6.0
7.0
8.0
9.0
10.0
11.0
12.0
13.0
14.0
Time, min

(C)

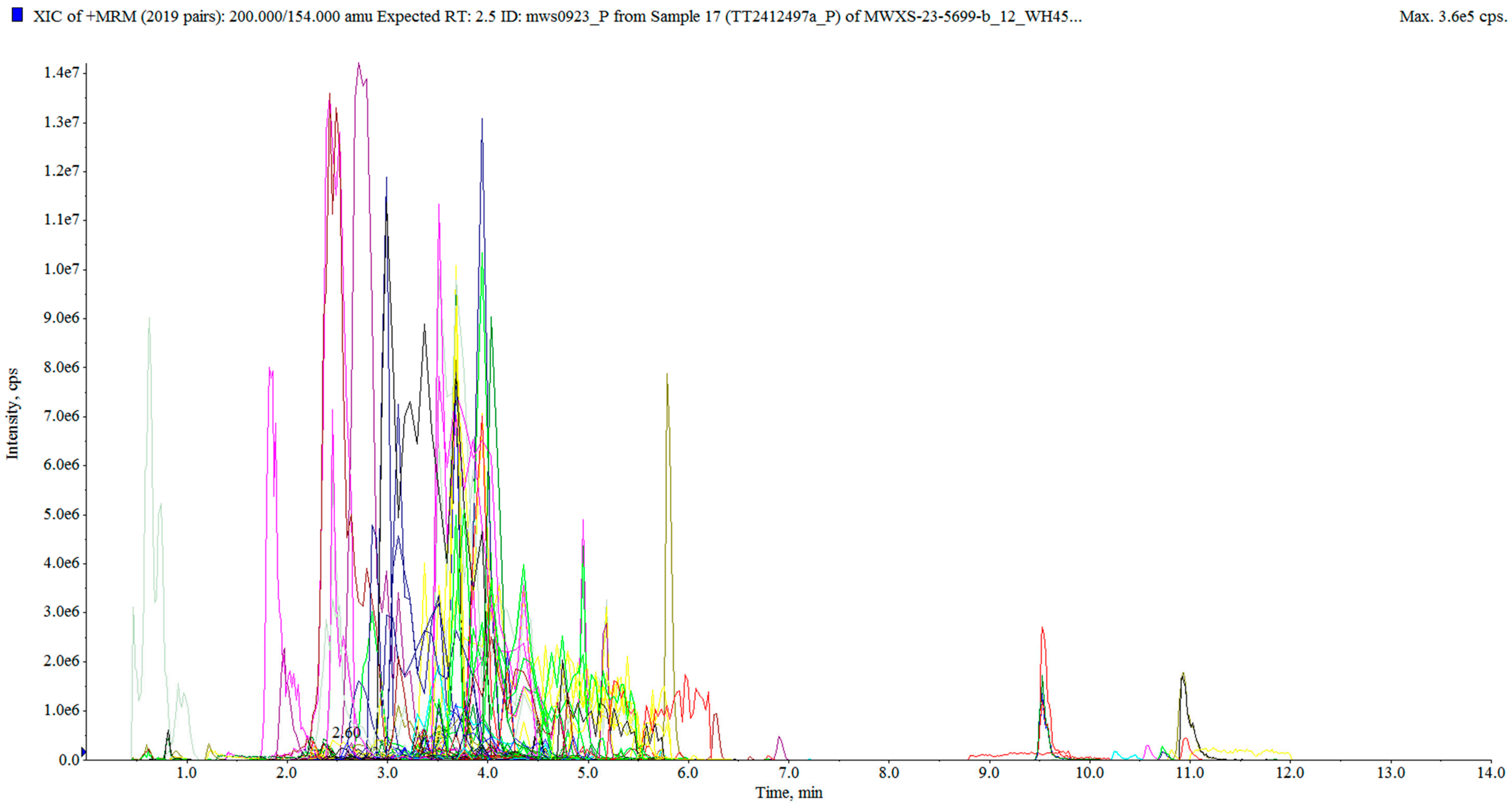

XIC of +MRM (2019 pairs): 200.000/154.000 amu Expected RT: 2.5 ID: mws0923_P from Sample 17 (TT2412497a_P) of MWXS-23-5699-b_12_WH45...
Max. 3.6e5 cps.
Intensity, cps
1.4e7
1.3e7
1.2e7
1.1e7
1.0e7
9.0e6
8.0e6
7.0e6
6.0e6
5.0e6
4.0e6
3.0e6
2.0e6
1.0e6
0.0
2.60
1.0
2.0
3.0
4.0
5.0
6.0
7.0
8.0
9.0
10.0
11.0
12.0
13.0
14.0
Time, min

(D)

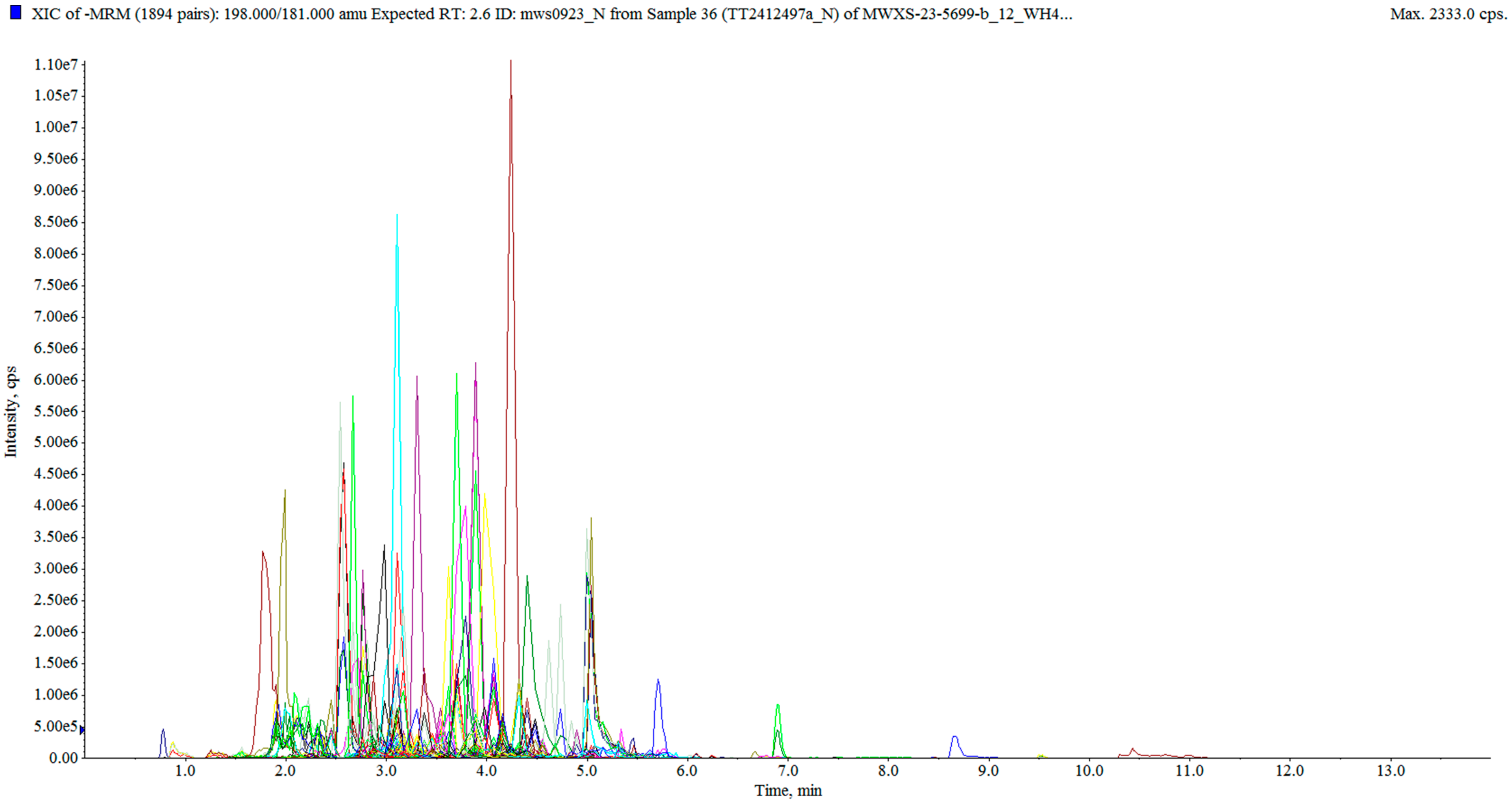

Figure. S1 MRM detection of multimodal maps. (A) MRM detection of VUFP multimodal maps-P; (B) MRM detection of VUFP multimodal maps-N; (C) MRM

detection of VUBP multimodal maps-P; (D) MRM detection of VUBP multimodal maps-N. P: positive ion mode; N: negative ion mode.
