# Supplementary material for: Identification and Analysis of Phenolic Compounds in Vaccinium uliginosum L. and Its Lipid-Lowering Activity In Vitro
Source: Foods. 2024 Oct 28;13(21):3438. doi: 10.3390/foods13213438 (PMC11545093; doi:10.3390/foods13213438)
Supplement: Supplementary file 1 [file foods-13-03438-s001.zip › Supplementary data Table S2.pdf]

Table S2. Anthocyanin monomeric substances in VUFP and VUBP

Number	Q1 (Da)	Q3 (Da)	Ionization model	Molecular Weight	Compounds	VUFP（μg/g）	VUBP（μg/g）
1	491.12	287.1	[M+H]+	491.1189512	Cyanidin-3-O-(6"-O-acetyl)glucoside	-	0.00701
2	611.2	287.1	[M+H]+	611.1612099	Cyanidin-3,5-O-Digalactoside	0.00891	-
3	727	287.1	[M+H]+	727.1721685	Cyanidin-3-O-(tartaryl)rhamnoside-5-O-glucoside	-	0.18938
4	535.1	287.1	[M]+	535.1087804	Cyanidin-3-O-(6-O-malonyl-beta-D-glucoside)	0.00754	0.01259
5	449.1	287.1	[M]+	449.1083865	Cyanidin-3-O-glucoside	147.16895	2.14704
6	611.2	287.15	[M]+	611.1612099	Cyanidin-3-O-sophoroside	0.05056	-
7	419.1	287.1	[M]+	419.0978218	Cyanidin-3-O-xyloside	10.73841	0.15781
8	449.1	287.1	[M]+	449.1083865	Cyanidin-3-O-galactoside	62.87949	-
9	611.2	287.1	[M]+	611.1612099	Cyanidin-3,5-O-diglucoside	0.07184	-
10	419.1	287.1	[M]+	419.0978218	Cyanidin-3-O-arabinoside	37.57671	0.27635
11	303.1	149	[M]+	303.0504777	Delphinidin	0.04408	-
12	435.5	303.1	[M]+	435.0927364	Delphinidin-3-O-arabinoside	-	0.51667
13	551.05	303.1	[M]+	551.103695	Delphinidin-3-O-(6-O-malonyl-beta-D-glucoside)	0.02103	-
14	799.23	303.1	[M+H]+	799.2296834	Delphinidin-acetyl-rhamnoside-rutinoside	0.02389	-
15	597.14	303.1	[M+H]+	597.1091743	Delphinidin-3-O-(6"-O-tartaryl)glucoside	0.02585	-
16	597.1	303.1	[M]+	597.1455598	Delphinidin-3-O-sambubioside	0.09842	-
17	465.1	303.1	[M]+	465.1033011	Delphinidin-3-O-glucoside	521.64312	7.07365
18	611.1	303.1	[M]+	611.1612099	Delphinidin-3-O-rutinoside	-	3.92845
19	465.1	303.1	[M]+	465.1033011	Delphinidin-3-O-galactoside	100.48002	-
20	935.2	303.1	[M+H]+	935.2457274	Delphinidin-coumaroyl-Sophorotriose	-	0.01197

Note: “-” means not detected in VUFP or VUBP.

Table S2. (Continued)

Number	Q1 (Da)	Q3 (Da)	Ionization model	Molecular Weight	Compounds	VUFP（μg/g）	VUBP（μg/g）
21	507.12	303.1	[M+H]+	507.1138658	Delphinidin-3-O-(6"-O-acetyl)galactoside	0.02200	-
22	627.17	303.1	[M+H]+	627.1349952	Delphinidin-3-O-(6"-O-caffeoyl)glucoside	0.03934	-
23	493.1	331.1	[M]+	493.1346012	Malvidin-3-O-glucoside	1971.48567	31.33085
24	625.19	331.1	[M]+	625.17686	Malvidin-3-O-sambubioside	0.34128	-
25	535.15	331.1	[M+H]+	535.1451659	Malvidin-3-O-(6"-O-acetyl)glucoside	0.03725	-
26	655.4	331.1	[M]+	655.1874246	Malvidin-3,5-O-diglucoside	0.57476	-
27	625.16	331.1	[M+H]+	625.17686	Malvidin-3-O-(6"-O-xylosyl)glucoside	-	0.02092
28	655.2	331.1	[M+H]+	655.1874247	Malvidin-3-O-glucoside-5-O-galactoside	-	0.02298
29	463.3	331.06	[M]+	463.1240366	Malvidin-3-O-arabinoside	144.41401	1.64463
30	697.2	331.1	[M]+	697.1979893	Malvidin-3-O-(6"-acetylglucoside)-5-glucoside	-	0.02187
31	595.14	271.1	[M]+	595.1662953	Pelargonidin-3-O-sophoroside	0.01311	0.01611
32	605.1	271.1	[M+H]+	605.1142597	Pelargonidin-3-O-(dimalonyl)glucoside	-	0.01161
33	433.2	271.1	[M]+	433.1134719	Pelargonidin-3-O-galactoside	0.39713	-
34	433.2	271.1	[M]+	433.1134719	Pelargonidin-3-O-glucoside	1.33492	0.02261
35	595.19	301.1	[M]+	595.1662953	Peonidin-3-O-sambubioside	0.14483	-
36	463.3	301.1	[M]+	463.1240366	Peonidin-3-O-glucoside	95.79673	1.45625
37	625.2	301.1	[M]+	625.17686	Peonidin-3,5-O-diglucoside	0.17888	-
38	433.2	301.1	[M]+	433.1134719	Peonidin-3-O-arabinoside	20.25373	0.17311
39	591.1	301.1	[M+H]+	591.1349952	Peonidin-3-O-(acetyl)(malonyl)galactoside	0.01682	-
40	609.16	301.1	[M]+	609.160816	Peonidin-3-O-(6-O-p-coumaroyl)-glucoside	-	0.01463

Note: “-” means not detected in VUFP or VUBP.

Table S2. (Continued)

Number	Q1 (Da)	Q3 (Da)	Ionization model	Molecular Weight	Compounds	VUFP（μg/g）	VUBP（μg/g）
41	433.11	301.1	[M+H]+	433.1134719	Peonidin-3-O-xyloside	-	0.04066
42	591.13	301.1	[M+H]+	591.1349952	Peonidin-3-O-(6''-O-acetyl-malonyl)glucoside	0.01749	-
43	625.16	317.1	[M+H]+	625.17686	Petunidin-3-O-(6''-O-rhamnosyl)-galactoside	0.00113	-
44	611.15	317.1	[M]+	611.1612099	Petunidin-3-O-sambubioside	0.02514	-
45	625.06	317.1	[M]+	625.17686	Petunidin-3-O-rutinoside	-	0.02912
46	449.1	317.06	[M]+	449.1083865	Petunidin-3-O-arabinoside	61.11889	0.64541
47	479.1	317.1	[M]+	479.1189512	Petunidin-3-O-glucoside	660.84982	6.56291

Note: “-” means not detected in VUFP or VUBP.
